# Integrating environmental physiotherapy into New Zealand undergraduate education: exploring current practice

**DOI:** 10.3389/fpubh.2024.1506697

**Published:** 2024-12-19

**Authors:** Olivia M. L. Stone, Katrina Bryant, Leigh Hale

**Affiliations:** Centre for Health, Activity, and Rehabilitation Research, School of Physiotherapy, University of Otago, Dunedin, New Zealand

**Keywords:** environmental sustainability, undergraduate education, physiotherapy, curriculum, holistic health, indigenous health

## Abstract

This paper describes the integration of environmental physiotherapy education into the physiotherapy curriculum in a New Zealand university in response to the environmental physiotherapy agenda and the University of Otago Sustainability Framework. We describe and discuss three learning activities, the associated challenges and lessons learnt, and the current position. Given the encompassing nature of environmental and health interactions, we aimed for multilayer immersive experiences using a range of pedagogical approaches. The first learning activity example exemplifies embracing and threading Aotearoa New Zealand’s indigenous knowledge and practices throughout our BPhty curriculum. The second example demonstrates how environmental physiotherapy can be made explicit within a delineated learning activity. In the third example, we describe a clinical placement learning activity that occurred in our student-led private practice. Recent full accreditation of the curriculum by the New Zealand Registration Board and positive student evaluations and feedback demonstrate that this integrated holistic curriculum is both acceptable and enjoyable. Frequent and rapid curriculum modifications in response to the COVID19 Global pandemic’s impact on teaching and learning have however prevented full formal curriculum evaluation at this stage. We envisage that this educational approach be an ongoing process of review and restructure. Aligned with global trends toward sustainability in healthcare, our goal is to prepare students to address the growing environmental influences on public health. By integrating environmental physiotherapy philosophy into the undergraduate physiotherapy curriculum, we aim to develop holistic healthcare perspectives in students that will strengthen future physiotherapy practice in New Zealand and internationally.

## 1 Introduction

### 1.1 Environmental physiotherapy

Planetary health is defined as “the health of human civilization and the state of the natural systems on which it depends” (1) pg. 1978, providing an overarching paradigm of the interdependence of human health with all elements of the ecosphere. Such a definition signifies the importance of inter-professional healthcare to address the intersecting global ecological crisis at play; this we acknowledge. In this paper, however we take a specific focus on Environmental Physiotherapy and entry-level physiotherapy education.

Environmental Physiotherapy is an emerging field within physiotherapy that focuses on the reciprocal relationship between human health and the environment. It emphasizes the impact of environmental factors such as air quality, access to green spaces, and climate change on individuals’ physical well-being and promotes an inclusive, more holistic view of physiotherapy within the umbrella concept of planetary health. Environmental Physiotherapy promotes social activism for sustainable healthcare that upholds environmental justice, considers multispecies impact and recognizes the symbiotic relationship of all life on the planet. This branch of physiotherapy recognizes that environmental factors can both positively and negatively influence health outcomes and aims to integrate environmental considerations into all aspects of the physiotherapy discipline (2, 3).

By incorporating environmental awareness into their practice, physiotherapists can provide more comprehensive care that addresses not only the immediate presenting issues but also the environmental factors that contribute to or influence them. Such an approach may be challenging to many physiotherapists as worldwide, current physiotherapy practice still largely subscribes to a reductionist, mechanistic biomedical / colonial model of health care (4). There is however progress toward more encompassing holistic viewpoints embracing concepts such as person-centered care (5) and bio-psycho-social approaches (2, 4). While critical physiotherapy philosophizes advanced by progressivist physiotherapy scholars are also, albeit reticently, being embraced by some in the profession (4), an ecological viewpoint that is more connected, dynamic and explores relationships beyond that of humanistic conceptions is still far from accepted usual practice (6).

Physiotherapy, as a health discipline, is well placed to become an essential component of world environmental health movement. Physiotherapists are trained to assess and treat the physical effects of environmental factors on individuals, including the health issues resulting from poor environmental sustainability. For example, managing respiratory conditions exacerbated by air pollution (7) or providing rehabilitation after injuries sustained during natural disasters (8–11), physiotherapists frequently work to mitigate the health impacts of environmental challenges. Physiotherapy interventions often involve promoting physical activity and outdoor exercise, which not only contribute to physical and mental health but also environmental stewardship (2, 12, 13). The move by the profession toward more holistic forms of healthcare that address not only the physical but also the social, emotional, and mental aspects of health, could make the intricate link between health and the environment more apparent. By including environmental awareness and reasoning into their practice, physiotherapists can promote preventive measures, support sustainable health and recovery, and mitigate environmental risks (14).

Although in its infancy, Environmental Physiotherapy (12) is now an accepted branch of physiotherapy that is becoming increasingly more influential in promoting increased education and environmental practice within the profession internationally. The Environmental Physiotherapy Association (EPA) was the first international network of likeminded physiotherapy clinicians, academics, students and supporters to focus specifically on advancing the field. The EPA provide resources and education, including guidelines for educators and institutions, as well as facilitating international collaborative research on planetary health and the role of physiotherapy practitioners in addressing environmental issues and planetary health (15).

The EPA introduced the Environmental Physiotherapy Agenda 2023 in 2020 with the unassuming goal:

*To ensure that every student beginning entry-level physiotherapy education from 2020 onwards will have education regarding the relationship between the environment, human health and functioning, and how this pertains to physiotherapy as part of their programme* (3) pg. 4.

### 1.2 Environmental physiotherapy education

Education that embodies sustainability, that teaches expectation of change and facilitates potential and capacity is essential (6). The foundation to instilling a sense of responsibility toward sustainable healthcare practices within the profession, probably lies at the stage of entry-level physiotherapy training. An ecological viewpoint and promoting environmental consciousness among physiotherapy students may pave the way for the next generation of physiotherapy practitioners to become holistic environmental champions and leaders of change.

Environmental Physiotherapy varies across cultures and countries, reflecting diverse environmental challenges and healthcare systems. For instance, in countries like Sweden, Norway and Denmark where access to nature is deeply ingrained in the culture, environmental physiotherapy emphasizes the therapeutic benefits of outdoor activities and wilderness rehabilitation (16–19). In contrast, in densely populated urban areas, environmental physiotherapy might focus on mitigating the health impacts, for example addressing the effects of air, water, noise and/or light pollution and promoting green infrastructure in urban planning (7, 20). Regardless of the context, integrating Environmental Physiotherapy education globally ensures that future physiotherapists are equipped to address environmental health challenges and advocate for sustainable solutions in their communities (21).

At time of writing, about 90 universities from across the globe have begun to integrate Environmental Physiotherapy into their programs, reflecting the growing international recognition for the need to address planetary health (3, 22, 23). The introduction of key concepts within environmental physiotherapy including, but not limited to, integrating indigenous knowledge, health equity, green space exposure and activity prescription, minimizing carbon footprints, exploring the United Nations Sustainably Development Goals (SDGs) and specific environmental factors (e.g., pollution and chronic obstructive airways disease) are underway (13, 23). The evidence showing that the profession and the educators are building momentum for change can be seen in websites, blogs and online activities (24, 25). For some key concepts, such as indigenous health and equity, details of how these are integrated into physiotherapy curricula are under investigation and emerging (26–28). However, for the larger field of environmental physiotherapy, curricula specific details are limited. Although examples can be seen on the EPA website as to how various universities are implementing environmental physiotherapy, they show minimal details. For example, Charles Sturt University (Australia) has integrated sustainability into its year 1, 2 and 4 courses, through scholarly review case-based scenarios and group work; and Neotia University (India) had integrated environmental physiotherapy throughout their year 4 curriculum. Cardiff University (Wales) website reports embedding sustainability into the physiotherapy curriculum with lectures, reflective learning and working groups including interdisciplinary collaborations (29) and the Monash University (Australia) website offers short courses in sustainable healthcare practice as the “first-of-its-kind…” (30). A review of Swedish higher education institutions that teach physiotherapy found that two of the eight institutions had yet to develop learning objectives that were sustainability focused. Only one institution had more than 5 % of their learning outcomes focused on sustainability and of the 1,255 total intended learning outcomes identified, only two were sustainably focused and related to hands on competencies (31).

While universities across the globe appear to be embracing Environmental Physiotherapy within their curricula, descriptions of how they are doing this, and the accompanying learning objectives and/or outcomes are not detailed or have yet to be published within the academic and peer-reviewed literature.

The purpose of this paper is thus to describe, at a granular level, the implementation of environmental and sustainable education into the physiotherapy curriculum in an Aotearoa New Zealand (NZ) university, the University of Otago. In this paper, we detail three examples of learning activities we have embedded into our entry-level Bachelor of Physiotherapy curriculum.

## 2 Environmental physiotherapy education within a New Zealand bachelor of physiotherapy curriculum

### 2.1 Background

At the University of Otago, School of Physiotherapy, we strive to prepare physiotherapy graduates that are not only prepared for current clinical practice in NZ but can be agile to respond to the future health needs of our population and future physiotherapy practice. We thus have a goal of ensuring our curriculum is forward-focused, exemplify contemporary teaching, and reflects our NZ indigenous identity. Traditionally our physiotherapy curriculum, like elsewhere in the world, was strongly influenced by the biomedical model, a model framed within scientific objectivity and reductionism (2). We have embraced in the last decade, the bio-psycho-social model. Influenced by the critical physiotherapy debate and our obligation and responsibility to NZ’s Te Tiriti o Waitangi, we are slowly progressing to post-colonial philosophies, including elevating Māori and Pacific People’s models of health and environmental health.

In the context of the current paper, we believe that physiotherapists need knowledge and skills to understand how environmental factors, such as pollution and climate change affect our patients’ health and recovery. By recognizing the importance of planetary health and demonstrating our commitment to holistic and sustainable healthcare, we aim to ensure that our curriculum is relevant and that our graduates lead the way in integrating environmental consciousness into patient care. Further to this, our University recently updated its *Tī Kōuka: the Sustainability Strategic Framework* (32). One pillar of this framework is *Te puaka tī, he tohu Raumati* - *Education for sustainability*. The whakatauki (Māori symbolic proverb) gifted for this pillar is “The flowering of the tī kōuka in spring is said to foretell the type of weather in the summer to follow.” This alludes to the importance of equipping our students and staff with the skills, understandings and values required to be more sustainable in their lives and work. It is how they act which will determine the sustainability of our long-term future.

Indigenous health philosophies can substantially contribute to the environmental health discussion. As a bicultural society, in NZ, significant value is gained from understanding Māori perspectives. In Te Ao Māori world view, there is no separation of the health (Hauora) of the whenua (land) and the health (Hauora) and wellbeing of humans. There is an intertwined, reciprocal relationship here. The Hauora of the whenua or papatūānuku (our mother) is carried with a strong sense of responsibility. We care for our mother, and she nurtures and provides for us. We do not own our mother; instead, we carry the responsibility of looking after the whenua for the perpetuation of our very existence as a species. Thus, good health comes from guardianship and nurturing of the whenua and waterways. Positive health and wellbeing are obtained and maintained from living off and with the provisions the natural world provides. In a practical sense, this includes maintaining healthy whenua and waterways as part of healthy exercise, therefore, ensuring that we are drinking and eating food, which is produced from healthy, sustainable environments. Thus, it is easy to understand the negative effects of colonialism and segregation from the environment has had on Hauora Māori.

Traditional Māori healthcare practices include Rongoā, a practice that incorporates the healthy balance of the mauri (the networks of the life forces found in the natural world). Knowledge of the balances that exist within the harmony of the natural world is encompassed with mātauranga Māori (Māori science/knowledge); the natural world balancing ecologies for supporting healthy life on earth are observed and described (33). Rongoā rākau, the use of herbal medicines from trees and plants is also well understood within mātauranga Māori. Māori have uncovered many natural medical properties, which are now being proven with western science techniques. This includes but not limited to, antibiotic properties in Manuka (34) and anti-inflammatory properties in Manu Tītī (mutton bird) spew (35).

In a country such as NZ, with a binding treaty between colonizers and indigenous peoples as a founding document (Te Tiriti o Waitangi), there is a responsibility to incorporate indigenous perspectives into health curricula and a physiotherapy registration requirement specifically addressing cultural safety and competency. As education providers, therefore, we have a duty of care to our people (tangata), our land (whenua) and for future generations. Māori perspectives of kaitakitanga are understood as being related to guardianship and protection of the environment. These are intimately entwined with wellbeing and longevity and the responsibility to establish good governance and continue this for future generations. This responsibility is specifically related to the health of the whenua and waterways. Culturally this connection is inseparable and is connected with the health of a person. For example, with water, Māori may ask who you are: “Ko wai koe?” (“What waters are you?”). This has many meanings; the waters of parents, the fact we are mostly water, what waters were your tupuna (ancestors) drinking and how healthy were they? The reference to water is deep throughout Māori philosophies and sense of identity. For example, the word for water spring, te Pū, and pool of water, Puna, are found in words for relations, such as mokopuna (grandchildren) or tūpuna (ancestors). Thus, environmental sustainability is an integral element when addressing physiotherapy approaches from a te Ao Māori perspective in NZ.

### 2.2 Pedagogical framework

In NZ and Australia, entry-level physiotherapy education is designed to produce competent, autonomous physiotherapists. The physiotherapy programs rely on Competency-Based Education and are designed to meet the competency standards set by regulatory bodies. In NZ, the registration authority is the Physiotherapy Board of NZ (PBNZ). The Physiotherapy practice thresholds describe the threshold competence required for initial and continuing registration as a physiotherapist in both Australia and NZ (36). To pass all prescribed courses and graduate with a Bachelor’s degree, we must evaluate that students meet these PBNZ set threshold competences. The curricula in physiotherapy programs in Australia and NZ, all of which are different, incorporate a mix of theory, knowledge, practical skills, and clinical experience to enable students to reach the threshold competencies for entry level registration. Whichever model/s of health each program subscribes to, physiotherapy is learnt experientially by active participation in learning activities that are underpinned by clear theoretical concepts (37).

The “Physiotherapy practice thresholds in Australia and Aotearoa New Zealand” were launched in 2015, with the thresholds competencies reflecting contemporary (at that time) physiotherapy and healthcare practice in Australia and NZ. The threshold competencies were arranged under key competencies within seven integrated roles of physiotherapy practice: (1) Physiotherapy practitioner, (2) Professional and ethical practitioner, (3) Communicator, (4) Reflective practitioner and self-directed learner, (5) Collaborative practitioner, (6) Educator and (7) Manager/leader. The thresholds have recently been updated and now stress the importance of cultural considerations and holistically delivered care, free of bias, discrimination and racism. Of relevance to this discourse, is that one threshold competency is:

“*Plan and implement an efficient, effective, culturally safe and responsive and client-centred physiotherapy assessment*,” of which one enabling component is “*recognise and evaluate the social, cultural, personal and environmental factors that may impact on each client’s functioning, disability and health*.”

The University of Otago’s Physiotherapy entry level program (BPhty), a four-year Bachelor’s degree program was revised from 2015 to 2018, guided by the Eberly Center framework for curriculum review and revision (38). This revision was in response to the 2015 launched registration threshold competencies as well as to the demands of the changing health service needs, government initiatives, and society demands. The aim was a curriculum that reflected and integrated NZ healthcare policies and prepared students for future practice and changing models of healthcare. NZ healthcare policies are increasingly focussed on person-centered and inter-professional care, equity and diversity, wellness, and management of individuals presenting with complex multi-morbidity (39). The curriculum revision resulted in an integrated curriculum, moving away from teaching content within the traditional siloed physiotherapy disciplines of neurology, cardiorespiratory and musculoskeletal, to an integrated multi-physiotherapy-disciplinary teaching approach (see Table 1). There was an emphasis on student centered learning, employing methods such as problem-based learning, case-based learning, reflective practice and self-directed learning (40).

**Table 1:** Outline of curriculum at a year 2 and 3 level: University of Otago physiotherapy specific papers taught as part of the BPHTY program.

Semester 1					
Year 2			Year 3		
Phty 254: Physiotherapy rehabilitation science 1		Phty 255: Physiotherapy clinical practice 1	Phty 354: Physiotherapy rehabilitation science 2		Phty 355: Physiotherapy clinical practice 2
Module 1 Foundations to physiotherapy practice	Unit 1: Welcome to the physiotherapy profession & to our program (Week 1)	Supervised clinical practice Integrated case studies Clinical Research	Module 1	Understanding Context Weeks 1 and 2	Supervised clinical practice Integrated case studies
	Unit 2: Understanding people and self (Week 2 to Week 3)				
	Unit 3: Understanding movement (Week 4 to Week 6)		Module 2	Prevention, Health/Wellness Week 3	
Module 2 Measurement and assessment	Unit 4: Understanding measurement (Week 7)				
	Unit 5: Understanding patient-centered assessment (Week 8 to Week 9)		Module 3	Primary care Weeks 4–13	
	Unit 6: Assessing posture and activity (Week 10)				
	Unit 7: Assessing body structure and function (Week 11 to Week 13)				

Semester 2						
Phty 254 Physiotherapy rehabilitation science 1		Phty 256 Physical activity in physiotherapy practice	Phty 255 Physiotherapy clinical practice 1	Phty 354 Physiotherapy rehabilitation science 2		Phty 355 Physiotherapy clinical practice 2
Module 3: Fundamentals of physiotherapy interventions	Unit 8: Basic / generic physiotherapy interventions (Week 1 to Week 5)	Topics covered: Importance of physical activity for health Tangata whenua approaches to physical activity Physical activity for Pacific populations Skills for teaching exercise Designing exercise programs Pharmacology - exercise	Supervised clinical practice Integrated case studies	Module 5	Tertiary care Weeks 1–5	Supervised clinical practice Integrated case studies
	Unit 9: Basic rehabilitation interventions (Week 6 to Week 9)	Exercise for balance strength, flexibility, aerobic function for clinical populations Contextual factors for physical activity participation Functional/Task orientated exercises		Module 6	Rehabilitation & community care Weeks 6–10	
	Unit 10: Principles of physiotherapy management (Week 10 to Week 12)	Biological adaptations to exercise Exercise induced hypoalgesia Exercise prescription following injury		Module 7	Professionalism & business Weeks 11–12	
	Unit 11: Clinical reasoning (Week 13)	Physical activity across the spectrum: Tertiary and secondary care; Primary care and community; Children; Older adults Hydrotherapy Strategies to promote physical activity uptake & motivation		Module 7	Module 7: Putting it all together and preparation for 4th year Week 13	

*Year 1 = First Year Health Sciences at University of Otago; Year 4 = a full-time clinical year plus research project; students also take papers in anatomy, physiology and pathology.

Our BPhty program also includes an Interprofessional Education component, aimed at familiarizing students with collaborative working, with ~900 students from nine health professional programs from the University of Otago and the Otago Polytechnic engaged at years 2 and 3, and multiple smaller groupings across the country working together clinically in their final years (41). Students learn alongside students from other health professions, an approach that prepares future health professionals for interdisciplinary healthcare delivery and thus overall improved health outcomes (42).

### 2.3 Incorporating environmental physiotherapy education

Our new curriculum design enabled incorporation of Environmental Physiotherapy learning objectives and activities, which we are gradually progressing. In addressing the aim of this paper, below we describe three examples of how we have done this to date. The first example exemplifies embracing and threading indigenous knowledge throughout our BPhty curriculum. The second example demonstrates now environmental physiotherapy can be made explicit within a delineated learning activity. In the third example, we describe a clinical placement learning activity that occurred in our student-led private practice.

#### 2.3.1 Authentic teaching of the Hauora Māori curriculum

Education about threshold competencies that enable effective and respectful interaction with Māori are incorporated at all levels of our BPhty curriculum. We endeavor to thread Hauora (health) Māori and the Principles of te Tiriti o Waitangi knowledge throughout the curriculum. We describe below the learning activities specific to the Hauora Māori curriculum as examples of how indigenous health and biculturalism can be included, recognizing that culturally, health of people (tangata), land (whenua) and waterways are inseparable.

In year 2 of the BPhty program (see Table 1), the teaching year starts with the module “Foundations to Physiotherapy Practice.” In unit 2 of this module is “Understanding People and Self,” and this unit includes the following learning outcomes:

Understand cultural safety in NZ:

- Self-awareness and self-reflection of impacts of practitioner cultural lens.
- Cultural knowledge and skill in cross cultural interaction.
- Understand and implement NZ policies that address equity (such as te Tiriti o Waitangi; He Korowai Oranga (Māori Health Strategy); He Kawa whakaruruhau ā matatau Māori (PBNZ’s Māori cultural safety and competence standard); and the United Nations Declaration of Rights of Indigenous People).”

To enable authentic teaching, the Hauora Māori learning activities are delivered for a full day at the Ōtākou Marae (the local meeting grounds, the focal point of the local Māori community. Māori see their marae as their tūrangawaewae - their place to stand and belong). The students learn about the Pōwhiri process (the formal welcome ceremony onto the marae) and tikanga (Māori custom and protocols), history and current application of the principles of te Tiriti o Waitangi and the impact of colonization on current Māori health status, Māori models of wellness and how to incorporate them into provision of physiotherapy services, importance of te reo Māori (Māori language), and the process of mihimihi statement of identity (who a person is: their mountain, river and people).

**Figure 1:**
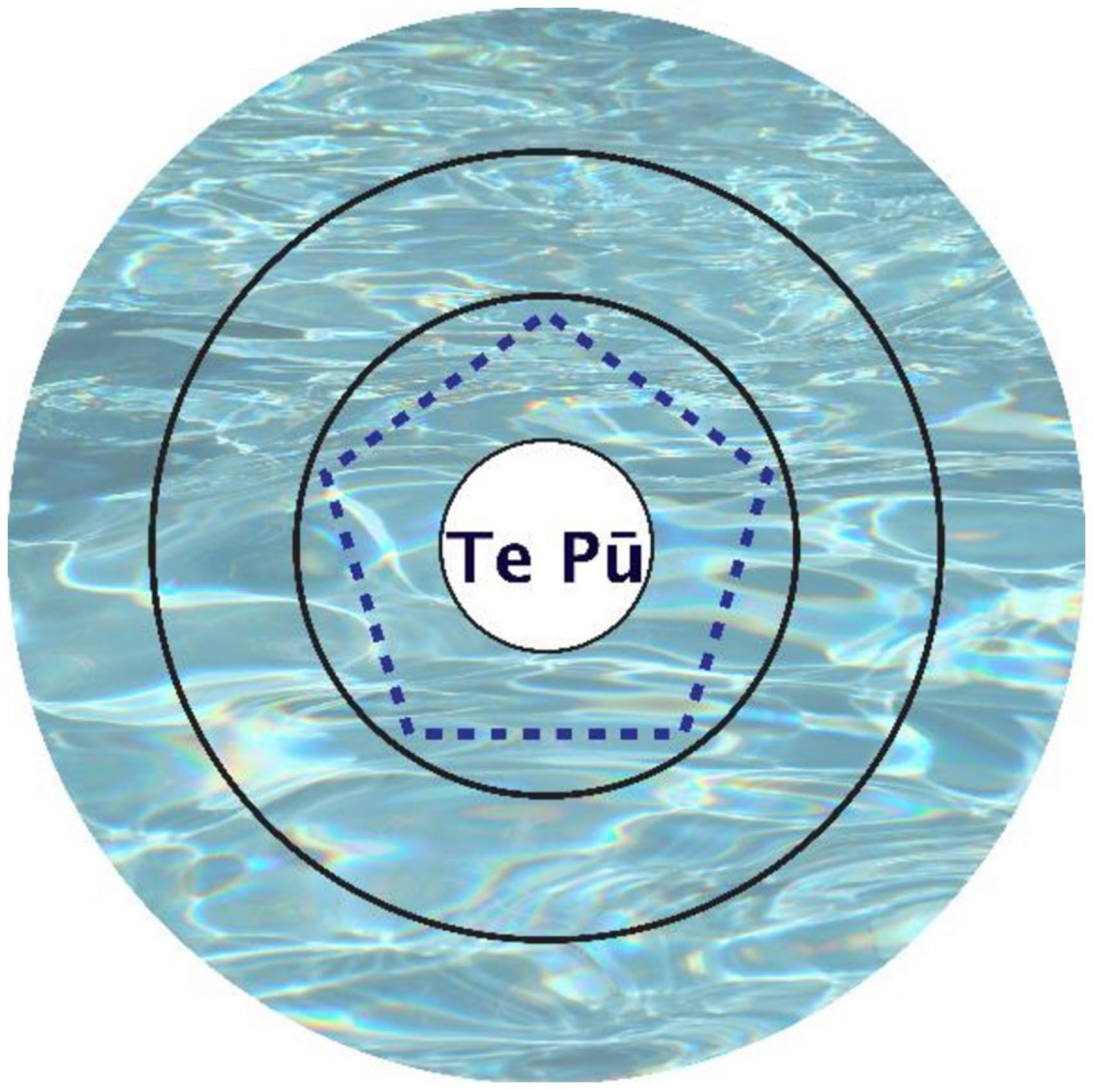
The Whakāro Pōkare visual tool.

In the fourth module of the year 2 course (“Assessment and measurement”) in unit 4 “Understanding patient-centered assessment,” all students, irrespective of culture, are taught the Whakaaro Pōkare Visual Tool (see Figure 1) developed by Pōtiki Bryant and Tikao to aid their patient interview process (43, 44). In the figure, the Pool signifies the patients’ life. Te Pū is the spring from the origin, signifying the patient (their essence). The lines signify the ripples of effect the patient has on their life. The pentagon shape reminds us that when we talk about life and wellness, there are many different aspects that make us well. The closer the aspects are to the source, the more important they are to the patient and their wellbeing. The visual model is laminated to enable white board markers to be used to record patient perspectives, which can then be photographed and wiped away at interview conclusion so that the laminated tool can be reused for another interview. Having the printed model laminated also provides an effective method for encapsulating a clear, visual depiction of the information from the patient, allowing the participant to see, manipulate and alter written information accordingly (43).

In year 3 of the BPhty program, the curriculum scaffolds onto intercultural competence and engagement with communities. Learning is via lectures, and workshops with activities with the following objectives:

(1) Intercultural competence: (i) Review the value and relevance in the physiotherapy context, (ii) Conduct a self-reflection exercise on own cultural background, and (iii) Consider how strategies for inter-cultural communication can be implemented.
(2) Engaging with communities: (i) Consider what constitutes ‘community’ and ‘engagement’, (ii) Consider effective engagement using community centered approaches and importance for physiotherapy within NZ, and (iii) Review outcomes of successful and poor community engagement.

In year 4 all students have a visit to the local marae at the city center in NZ they have been allocated to for their final year, a year mostly comprising supervised clinical practice. Marae visits include undertaking the pōwhiri process in a healthcare setting, and learning Māori movement practices in rehabilitation, Rongoā, and Taurite Tū (a Māori strength and balance exercise program).

#### 2.3.2 The Dragon’s Den physiotherapy Aotearoa - developing a modified business plan for a physiotherapy practice with a social mission

In the “Business module” of the Year 3 paper (see Table 1), students work in groups on an assignment “*The Dragon’s Den Physiotherapy Aotearoa* - *Developing a modified business plan for a physiotherapy practice with a social mission*.” Each group must develop a modified business plan for a new physiotherapy practice which has at its heart a social mission. Groups are allocated the location (i.e., a NZ region or town) for their new practice. Each group then sell their idea to the “Dragons” in the “Dragon’s Den Physiotherapy Aotearoa” session. Among several objectives of this assignment are the following: (i) understand local responsibility for contributing to global and local sustainable development; (ii) apply the concepts of professional duty and social responsibility; and (iii) apply basic principles of private practice management, including the development of business plans, sustainability, equity, diversity, accessibility and marketing services. During their “Dragon’s Den” styled presentation, groups are marked on their proposed physiotherapy practice’s social mission and vision, plans to enable equity of service and accessibility for all, and how they intend to offer sustainable physiotherapy (in the context of climate change). Students have preparation lectures on “The Application of Sustainability” and the United Nation’s Sustainable Development Goals (SDGs), and are directed to explore the Environmental Physiotherapy Association website^1^ and the University of Otago’s Sustainability Framework (32).

#### 2.3.3 Student-led clinics

In the final clinical year of the BPhty program many students are placed in one of the two School of Physiotherapy primary care private practice student-led physiotherapy clinics. These clinics offer physiotherapy services to the public, allowing fully supervised physiotherapy students to provide physiotherapy services while completing clinic placement and learning pseudo-autonomous practice. With the help of students and staff, the smaller of these facilities began the process of critically examining their processes and functions in line with the SDGs. Postgraduate students, undergraduate clinic based students and all staff in the School of Physiotherapy (*n* = ~90) were invited, via email, to provide feedback on “what concepts, thoughts or philosophies spring to mind when considering the SDGs,” to assist with inspiration for building a starting platform. They were provided with a description of each SDG and asked to record thoughts and ideas relating to physiotherapy and specifically to “think of the SDGs as inspirations…” Collectively we received more than 100 responses with least one idea was raised for each of the SDGs, the five most popular SDGs producing more than 50 suggestions (see Table 2). These data were then later shared in as an education session with staff. While primarily used as a student learning activity, a secondary objective was to guide the clinical facility to become a more sustainable healthcare practice and an immersive student experience. Many current clinic protocols were identified as already addressing SDGs and new protocol ideas generated. Following this exercise, multiple new initiatives and modifications were introduced, with many proving to be more efficient than current practices (see Table 3 for detail).

**Table 2:** Top five sustainable development goals (SDG) to improve the clinical teaching facility as suggested during consultation of staff and students (*n* = ~90).

		SGD	Examples of suggestions
Ranked according to number of suggestions	1	Goal 3: Ensure healthy lives and promote well-being for all at all ages	“Free online classes that do not require any equipment. Promoting exercise in outdoor environments…” “More funding for pediatric physios, increase screening of children” “Health promotion events such as cross-generational activities – promote walking routes that are accessible to all…”
	2	Goal 4: Ensure inclusive and equitable quality education and promote lifelong learning opportunities for all	“Providing easily accessible, free content related for the general public to empower them to look into questions they may have themselves. “ “Educating patients to self-manage and understand own condition”
	3	Goal 1: End poverty in all its forms everywhere	“Provide telehealth options as a full-time service for those who struggle to access due to cost of travel, time off etc.” “More subsidies for low-income families to attend physio.”
	4	Goal 5: Achieve gender equality and empower all women and girls	“Content for women led by women. Making all genders aware of common problems they and other genders may face. Including all genders not just the stereotypical two. Multi-gender facilities for the community to use in practice.” “Giving all students equal opportunities, regardless of gender and/or background.” “Addressing/ acknowledging unconscious bias” “Encourage inclusiveness of gender diversity at all levels of university including admission to courses, research, and other university policies”
	5	Goal 6: Ensure availability and sustainable management of water and sanitation for all	“Water bottle filling stations and water filtering stations.” “Water cooler in the clinic, a few sanitary supplies in bathrooms” “Minimize unnecessary water use”

**Table 3:** Protocols used within the clinical teaching facility.

Initiative or modification	Status	Description and/or points of interest	Applicable SDG
Unisex toilets	Existing	Toilets and showers were non-gendered. Allowing access to all.	5, 10, 16
Provision of fresh drinking water	Existing	Filtered water was provided to all staff and visitors.	6
Biodegradable cups, made from sustainably harvested forests	New	Although the capacity to have reusable cups existed, concerns over infection control during the COVID 19 Pandemic and increase in use detergent and electricity meant we changed to sustainable biodegradable cups.	12
Students were surveyed for sustainable suggestions	New	In the initial stages, this was to engage students in the change process and elicit sustainable critical thinking.	11, 12, 13
“Green” suggestion box was placed in the waiting room for feedback from staff, students and public.	New	Most comments related to transport to and from the physiotherapy service, which we were unable to address at the time. Namely, the provision of safe bike stands and most consistently that people would pay for parking, if the money raised was used to plant trees to combat the environmental cost of using motor vehicles.	11, 12, 13
Plants	Existing	Students seemed to enjoy the environmental theory as a result they presented the clinic staff with additional plants to “green the space” upon finishing their placements.	3, 15
Introduction of Telehealth	Existing	Telehealth is known to address inequities by promoting access.	3, 10, 12
Introduction of card access photocopying	New	This significantly decreased the paper consumption. Potentially due to the extra time required and number of steps involved. E.g., Staff preferred to read from screens rather than log in and select options to print and then, having walked to the copier, potentially waiting in line to log in to the printer, then again selecting options before collecting their printing.	12
Introduction of digital signing of client/health paperwork.	New	This enabled forms to be completed without printing paper (the majority of which had been later scanned back to digital form and shredded)	12
Digitisation of student paperwork	New	All paperwork was digitized, and a new shared secure server was set up so that all paperwork could be completed and accessed by staff and students electronically. This increased efficiency within the clinic.	12
Lamination of all required non-digital paperwork	New	Laminated paper can be digitised for storage after use, and then wiped clean for the next user.	12
Ensure the clinic was ethnically inclusive			3, 10
Bilingual signage	Existing	e.g., directions within the facility	3, 10
Atmosphere	Existing	Inclusion of culturally significant indigenous items: e.g., the clinic hung Māori flax weavings that had been created by university staff and were part of the clinic heritage.	3, 10
Interpretation services	Existing	Interpretation services were accessible via a telephone service.	3, 10
Removal of unnecessary linen	New	The cost environmentally of cleaning sheets, pillowcases and towels after each client, in energy consumption and chemical waste was considerable. Instead, disinfecting the surfaces between clients proved not only more effective as we approached the COVID pandemic, but also financially astute.	12
Encouraging outdoor exercise	New	The teaching facility, although located in the CBD was less than 50 m from a riverbank. Staff and students were encouraged to take their clients outside were appropriate for assessment and/or treatment.	3, 15
Encouraging walking meetings	New	Where feasible, staff and students were encouraged to undertake “walking meetings.” This was the preferred option for all one to one meetings or meetings perceived to be more stressful or requiring privacy. E.g., providing feedback on student performance.	3, 15
Lunchboxes	New	Staff and students were asked to bring their food renewable receptacles, to minimize food wrapping waste.	12

## 3 Reflection and discussion

In this paper, we approach planetary health with a simplistic view, of encompassing the health of our planet, including human civilization, and the complex, interdependent interactions between the two. We consider Environmental Physiotherapy a focused subset of planetary health that explores the specific role of the physiotherapy profession within this broader concept. While our paper focuses specifically on Environmental Physiotherapy, we recognize that meaningful, sustainable change in this area cannot occur within a single profession and thus effective responses to planetary health challenges require interprofessional collaboration. While interprofessional education is a core part of all health professional programs at the University of Otago, as mentioned earlier, this paper was limited to describing the integration of Environmental Physiotherapy into our BPhty program, illustrated by the description of three novel learning activities.

Although we successfully incorporated Environmental Physiotherapy learning activities into our revised BPhty curriculum, we unfortunately have not been able to formally evaluate the outcomes of these initiatives. In revising our curriculum, we initiated the Continuous Feedback model (40) as a means of ongoing evaluation and refinement, but this process was disrupted by the COVID pandemic. Due to the pandemic, the curriculum underwent multiple adaptations to respond to the NZ government’s many, and often prolonged, mandated lockdowns over a two-year period. Adaptions included changing the sequence of delivery of the curriculum (e.g., teaching all the theoretical components online for about 10 weeks and then teaching the in-person practical and workshop components in the subsequent semester of 13 weeks with strategies for social distancing and smaller classes, using telehealth in clinical placements). As the students moved up a year, the curriculum had to again be adapted to cover what had/had not been taught in the previous year. This caused a major disruption to the sequencing of our revised curriculum, and it became impossible to compare data from subsequent years. Further, due to the financial recession caused by both the COVID Pandemic and the two devastating cyclones that hit NZ in 2023 (45), the smaller clinic was closed and the immersive experience ceased before we were able to review and assess the outcome. Our curriculum was however reaccredited in 2023 by the PBNZ and gained full accreditation with no conditions for 7 years, indicating that our curriculum and approach to teaching (including our novel environmental learning activities) were acceptable and our new graduates able to automatically register to practice physiotherapy in NZ and Australia. Anecdotal evidence suggests the students liked the Dragon’s Den initiative and presented innovative ideas for physiotherapy private practice. The marae visits to in years 2 and 4 usually gain favorable student evaluations. Students also enjoyed the sustainable clinic concept (always keen to pick up new ideas, e.g., no more plastic cling wrap at lunch) and were passionate about sharing their ideas for improving the sustainability via the drop box located in the clinic reception. Perhaps future educational endeavors could use the PIER (Planning, Implementation, Program Evaluation, Program Revision) Framework to evaluate the integration of environmental physiotherapy teaching activities into the clinical practice setting (46). Evaluating outcomes of such endeavors probably leans toward qualitative methodologies to enable a deeper, more nuanced understanding of success and ways to improve than would blunt instruments such as surveys or student grades.

### 3.1 Practical implications, objectives and lessons learned

Incorporating environmental health and sustainability into the competency standards is a step toward preparing future physiotherapists to address environmental determinants of health. Providing a learning environment that allows students to experience integrating sustainable practices into simulated or real clinical care is logical and congruent with international movements and calls to action (2, 20). Environmental Physiotherapy teaching activities can easily be incorporated into theoretical and practical components of the curriculum, and many resources are readily available, although can have a substantial step-up cost, such as telehealth (47).

When, in early 2020, NZ went into lockdown during the first wave of the COVID-19 pandemic, telehealth facilities in our School’s clinics became not only the primary source of patient interaction but also how the students were taught during their clinical placements. A global pandemic exemplifying the impact of nature on humanity also inadvertently supported the telehealth integration into teaching. For the first time, students completed entire six-week clinical placements, working with patients and supervisors without ever leaving their homes. The same software (Zoom Videoconferencing Software) that allowed physiotherapists to treat their clients online also allowed students to see their patients with supervisors actively involved in and controlling the teleconference clinical sessions. Initially an initiative aimed at allowing equitable access for those patients who could not attend the clinics, it became the only available option, thus validating its importance. With video communication, teleconferencing and mobile phones now common place globally, this is recognized as a cost-effective initiative for the client.

In our experience, integrating new sustainable technologies with existing healthcare infrastructure can be complex and costly, as can ensuring compatibility between old and new systems. For example, the equipment (technology) required to reduce our clinic’s paper consumption, such as log-in facilities on new smart printers, tablets, and tablet accessories like digital pens, are all expensive items. It is reasonable to expect that most clinical environments would need to introduce such measures gradually due to inhibitory costs. Surprisingly, the amount of printing significantly declined with the required log-in alone.

Finding sustainable alternatives that meet healthcare standards can be difficult. Additionally, sustainable products may have higher costs or limited availability. For example, on a very basic level, biodegradable cups from sustainably harvested forests are inevitably more expensive than regular plastic cups. Alternatives like glass cups, although slightly more expensive initially, are reusable. However, in a healthcare setting, considerations about sterilization, made more prominent during the COVID pandemic, can be equally frustrating. The balance between maintaining infection control and implementing eco-friendly practices is at the forefront of our planning.

While the transition to sustainable healthcare systems is essential, the economic, technological, and logistical complexities are challenging; we found a focus on continuous improvement and stakeholder engagement to be a valuable resource in our sustainability efforts. Unfortunately, the unforeseen closure of the facility prevented us from empirically assessing the efficacy of the protocols introduced in this initiative. Nevertheless, the process effectively served as a pilot study.

Given the strong acceptance of the sustainable initiatives introduced in the student-based clinic, we believe many of these practices could feasibly be expanded across the entire School of Physiotherapy. We recommend starting with smaller, manageable goals, such as discouraging the use of soft plastics in lunches and waste by encouraging staff to adopt renewable, reusable containers. Additional steps could include ensuring only reusable cups and utensils in the staff room and promoting “walking meetings” to further integrate sustainability into daily routines.

Due to the smaller size of the clinic, we had the opportunity to implement targeted initiatives that resulted in tangible, immediately noticeable changes for both students and staff (Table 2). In contrast, larger institutions, forced to cover a much wider scope (such as teaching hospitals and universities), often need to begin with broader, more abstract measures, such as forming committees, developing policies, or drafting charters (32, 48, 49).

Our School’s acknowledgement, and weaving in, of Hauora Māori and the Principles of te Tiriti o Waitangi knowledge throughout the curriculum is a work in progress as our levels of understanding of what this means in Physiotherapy and for the health outcomes of the people of NZ deepens. This is compulsory education for all healthcare professionals in NZ. Cognisance must all be given to Māori students’ knowledge, as this is their heritage and way of being, and thus we must strive to extend their knowledge and practice. We continue to promote the understanding that the client / patient belongs to a whānau, community, culture and respect the connection between physical, mental, spiritual, and environmental health. Ultimately, we need to constantly work toward a shift from a colonial style of teaching and the exploitation of people and nature and environmental degradation (50). As health professionals, we must promote a move away from the degradation of culture and our living environment that impacts on all species especially humans. This logic has started conversations and debate about underpinning from the start, our BPhty curriculum with the knowledge and understandings of Hauora Māori and Māori models of healthcare. Then only later in the program introducing concepts developed in other parts of the world, such as the International Classification of Functioning, Disability and Health, to enable global citizenship. This example, while directly relating to NZ, gives food for thought to other nations on how they can adapt their physiotherapy curricula to their indigenous philosophizes.

### 3.2 Looking forward

With the need for sustainable healthcare urgent and the global impact of health care being a significant contributor to environmental degradation, further ways of how to integrate planetary health and environmental sustainability into physiotherapy curricula are undoubtedly forthcoming. Until such a time we can endeavor to:

*To inspire multi-level leadership and collaboration; empower and privilege students’ voice; develop a curriculum framework with a planetary health lens, provide professional development for educators… and finally integrate* [Sustainable Healthcare Education] *into course accreditation standards…* (51) pg. 331.

World Physiotherapy’s Physiotherapist Education Framework notes that a graduate should be competent to practice “independently, in a safe, effective, equitable, accessible, sustainable and ethical manner” (52), although it does not directly address environmental sustainability. World Physiotherapy’s Guidance Document for the development of physiotherapy curricula, however notes that a curriculum “should be situated within a broader context such as human rights, Sustainable Development Goals (SDGs), climate crisis, public health, and health promotion” (53).

As an international profession, we should aim to model a more holistic approach with greater understanding and collaboration from indigenous cultures that have sustainably maintained land and ecosystems. It is important to emphasize evidence-based practice in environmental health to ensure that students are capable of critically evaluating and researching the health impacts of environmental factors, likely to become more prominent in future healthcare. The movement toward sustainability must be supported by educational institutions and applied homogenously across all levels from top academics to first year students (54, 55) so that all health practitioners, both established and future, innately advocate for sustainable practices in their professions. Educational policies and frameworks, and institutional directives that command these are difficult to influence and difficult to change (56). With persistence, however, the planetary health and sustainable healthcare movement is gaining traction and integrating sustainability into healthcare education is happening across the globe (56, 57). We hope this paper has provided some tangible ways of how to incorporate such education into entry-level health professional programs.

## Data Availability

The original contributions presented in the study are included in the article/supplementary material, further inquiries can be directed to the corresponding author.

## Footnotes

^1^ https://environmentalphysio.com
